# LAPKaans: Tool-Motion Tracking and Gripping Force-Sensing Modular Smart Laparoscopic Training System

**DOI:** 10.3390/s20236937

**Published:** 2020-12-04

**Authors:** Luis H. Olivas-Alanis, Ricardo A. Calzada-Briseño, Victor Segura-Ibarra, Elisa V. Vázquez, Jose A. Diaz-Elizondo, Eduardo Flores-Villalba, Ciro A. Rodriguez

**Affiliations:** 1 Tecnologico de Monterrey, Escuela de Ingeniería y Ciencias, Monterrey, Nuevo León 64849, Mexico; A01230117@itesm.mx (L.H.O.-A.); A01330981@itesm.mx (R.A.C.-B.); victor.segura@tec.mx (V.S.-I.); elisa.vazquez@tec.mx (E.V.V.); 2 Laboratorio Nacional de Manufactura Aditiva y Digital (MADIT), Apodaca, Nuevo León 66629, Mexico; 3 Tecnologico de Monterrey, Escuela de Medicina y Ciencias de la Salud, Monterrey, Nuevo León 64710, Mexico; jadiaze@tec.mx

**Keywords:** laparoscopic surgery, surgery simulator, motion tracking, force sensor, additive manufacturing

## Abstract

Laparoscopic surgery demands highly skilled surgeons. Traditionally, a surgeon’s knowledge is acquired by operating under a mentor-trainee method. In recent years, laparoscopic simulators have gained ground as tools in skill acquisition. Despite the wide range of laparoscopic simulators available, few provide objective feedback to the trainee. Those systems with quantitative feedback tend to be high-end solutions with limited availability due to cost. A modular smart trainer was developed, combining tool-tracking and force-using employing commercially available sensors. Additionally, a force training system based on polydimethylsiloxane (PDMS) phantoms for sample stiffness differentiation is presented. This prototype was tested with 39 subjects, between novices (13), intermediates (13), and experts (13), evaluating execution differences among groups in training exercises. The estimated cost is USD $200 (components only), not including laparoscopic instruments. The motion system was tested for noise reduction and position validation with a mean error of 0.94 mm. Grasping force approximation showed a correlation of 0.9975. Furthermore, differences in phantoms stiffness effectively reflected user manipulation. Subject groups showed significant differences in execution time, accumulated distance, and mean and maximum applied grasping force. Accurate information was obtained regarding motion and force. The developed force-sensing tool can easily be transferred to a clinical setting. Further work will consist on a validation of the simulator on a wider range of tasks and a larger sample of volunteers.

## 1. Introduction

Laparoscopic surgery was originally intended as a diagnostic approach back in the 1960s; however, it has evolved to become the gold standard for many procedures, with nearly 15 million worldwide laparoscopic surgeries performed each year, due to the overwhelming benefits when compared to open surgery [1,2,3,4]. Surgeons, requiring only small incisions (5–12 mm), introduced long and narrow instruments through a narrow port (trocar) along with a camera into the abdominal cavity. They would navigate instruments on a screen using live video of anatomical structures. Consequently, laparoscopic surgery offers reduced periods of hospitalization, post-operative pain, recovery time, and costs, along with better functional and cosmetic results [5,6,7].

While greatly beneficial to the patient, proper dominance of laparoscopic surgery techniques requires a long steep learning curve. The most common drawbacks encountered are loss of spatial perception, inversion and scaling of movements, loss of tissue sensitivity, and perception of counteracting forces. They are mainly generated by torque and friction created at the access points [8,9], known as the “fulcrum effect” [10,11]. These factors may lead novice surgeons to take subjective decisions, resulting in wrong executions or involuntary movements, which could lead to unanticipated complications [12]. Demi et al. demonstrated that manipulating tissue without sensitive feedback increases the risk of tissue damage [8]. Similarly, Tavakoli et al. proved that this may lead to avoidable, long surgeries [13]. Furthermore, complications, such as bleeding or unintended perforation, may force the surgeon to convert the originally laparoscopic surgery procedure into an open surgery, increasing patient mortality [14]. Some studies have demonstrated that around 1.1% of cholecystectomies resulted damage to bile ducts [15,16], with the lack of tactile experience as the main contributing factor [17]. Further studies found bowel injury in nearly 18% of laparoscopic procedures with a related 3.6% mortality rate [18,19]. Although occurrence is low, damage must be prevented. Therefore, a significant amount of training and practice is required to master these techniques, avoiding complications, and decreasing the learning curves [20,21].

Virtual reality and augmented reality-based simulators are capable of reproducing complete digital laparoscopic surgeries [8]. Virtual simulators have shown surgical skill improvement due to the surgeon’s interaction with realistic scenarios [22,23]. They also provide feedback in terms of procedure execution time and quantify the distance traveled by the instruments. However, the high cost and bulkiness associated with virtual simulators are the main factors that limit their widespread use in resource-limited centers and medical schools [7,24,25]. On the other side of the spectrum, a simple training box with a webcam inside is often used to develop basic skills in handling laparoscopic surgery instruments. These kinds of box trainers can only provide visual feedback to the surgeon. Therefore, medical schools have identified the need to develop a simulator that strikes a balance, providing quantitative and meaningful feedback (beyond the box trainer capabilities) to the surgeon in training, but avoiding the high cost of full-blown virtual surgery simulators.

We present LAPKaans, from the word “laparoscopy” and the Mayan word “kaans”, which means “to instruct”. LAPKaans is a smart laparoscopic training box equipped with different modules: Tool-motion Tracking, Grip Force Sensing, Force Training, Data Acquisition and Processing, Graphical User Interface, and Training Tasks. It was designed to be inexpensive, portable, user-friendly, and compatible with both: (a) standard laparoscopic instruments and (b) exercises from The Fundamentals of Laparoscopic Surgery (FLS), additional to other tasks reported by previous investigators [26,27]. Commonly used sensing technologies, components, and materials were employed, without limiting the functionality, ergonomics, or shape of standard surgical instruments.

## 2. Materials and Methods

### 2.1. Tool-Motion Tracking Module

**Figure 1:**
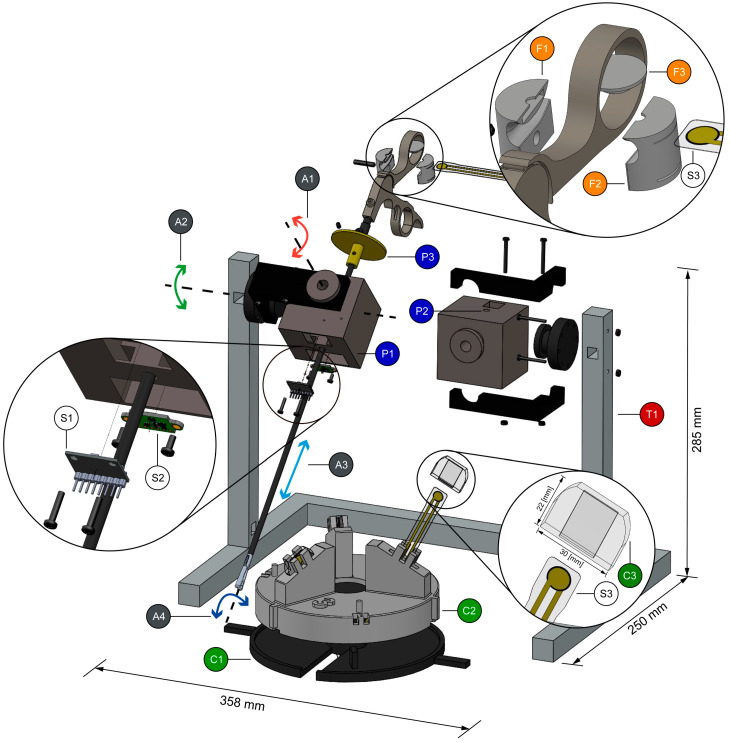
Explosion view of LAPKaans smart laparoscopic training system featuring 4 degrees of freedom (A1–A4). Tool-motion tracking (P1–P3), Gripping Force Sensing (F1–F3), and Force Training (C1–C3) modules mounted on the aluminum frame (T1) with the position and force sensors (S1–S3) are shown.

The exploded view of the LAPKaans smart laparoscopic training system and modules are shown in Figure 1. The system dimensions were designed to be compatible with an ETHICON basic tasks training set (Johnson & Johnson^®^, New Brunswick, NJ, USA) with similar dimensions as other trainers in the market. The Tool-Motion tracking module measures three-dimensional position at the tip of the instrument for further measurement of the path length during execution and additional tool-speed computing. In related studies, this metric is associated with the surgeon’s skills. Only a minimal modification to standard laparoscopic instruments was introduced in order to take advantage of inherent instrument’s complexity, functionality, and ergonomics. As seen in Figure 1, it consists of two instrument guides, emulating a trocar (P1) (in the next sections they will be referred to as smart trocars or trocars). They are three-dimensional (3D)-printed by Fused Deposition Modeling (FDM) made from Polylactic Acid (PLA) mounted in an aluminum support (T1). A mechanism like the Immersion Virtual Laparoscopic Interface (Immersion Medical, Gaithersburg, MD, USA) is proposed. The system features 4-degree of freedom (DOF): yaw (A1) and pitch axis (A2); translation (A3) through the insertion point (P2); and roll axis in the instrument shaft (A4). The trocar itself is cubic with a centered hole (P2) for the insertion of standard laparoscopic instruments. Inside the trocar, there are compartments for position sensors and wires hidden from the user, avoiding any movement restrictions.

Two sensors are required for each smart trocar. The trocar orientation is achieved using a small 6-axis motion tracking device MPU6050 sensor (S1) (InvenSense, San Jose, CA, USA), which features a gyroscope and an accelerometer. A linear position sensor (S2) acquires the tool-insertion-depth: VL53L1X (STMicroelectronics, Plan-les-Ouates, Switzerland). This Time-of-Flight (TOF) sensor requires a target with a flat surface to bounce the emitted pulse. Thus, a detachable disk-shaped piece (P3) was 3D-printed and placed at the upper part of the instrument’s shaft. The disk is fixed with an oppressor to prevent inaccurate measurements due to its movement. Nevertheless, if a change in distance from the sensors is done during the setup, this variation will be detected at the initial calibration process (Section 2.4).

### 2.2. Gripping Force Sensing Module

The proposed training system includes force feedback with the application of an assembly sensor (Figure 1(F1–F3)). It indicates the surgeon in training the approximate grasping force being applied at the tip of the surgical instrument, by measuring the force exerted at the handle. Two 3D printed parts attached to the tool’s handle (F1–F2) contain a force sensor that measure the applied handle force transmitted through a button (F3). A functional prototype of the handle adaptor was achieved through additive manufacturing: FDM with Polycarbonate (PC-ISO) through a Stratasys Fortus 400 machine. The handle adaptor is instrumented with a FlexiForce thin-film force sensor (S3) (Tekscan, Boston, MA, USA).

### 2.3. Force Training Module

The functionality of the proposed training system is further expanded with a force training system module (Figure 1(C1–C3)). This system includes three thin-instrumented polydimethylsiloxane (PDMS) phantoms (C3) with varying degrees of stiffness and a rotating carousel (C1–C2) for their management. The instrumentation is done with the same force sensors (S3) applied in the gripping force sensing module (Section 2.2). Thin-film sensors are embedded in the internal cavity of each phantom. The phantoms emulate different tissue stiffness, ranging from soft (i.e., adipose tissue) to firmer organs, such as the liver or kidneys. PDMS is an elastomer with an elastic modulus of 1 MPa, transparent, and low cost. PDMS mechanical properties can be modified with three main parameters: base to cross-linker agent concentration, curing temperature, and curing time as described by Seghir et al. [28], thus, providing a vast stiffness profile.

For this purpose, phantoms were manufactured with base to cross-linker ratios 10:1, 2:1, and 19:1 for hard, medium, and soft, respectively. PDMS curing was done at 100 °C for 2 h. The instrumented phantoms were gripped 10 times with the gripping force sensing module to detect the differences in stiffness by plotting the gripping force compared to the force measured at the phantom itself. Further analysis considers the different combinations of phantoms positions around the carousel since they can be rearranged.

### 2.4. Data Acquisition and Processing

**Figure 2:**
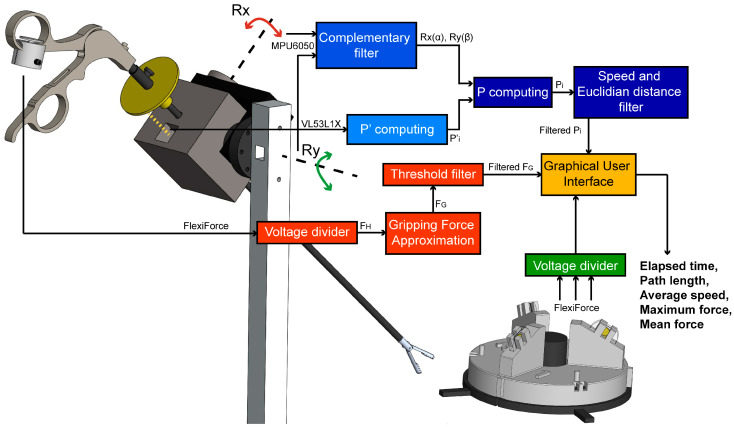
Graphical flowchart of the measurement computing. The tool-motion tracking module is depicted in blue, the gripping force sensing module in orange, the force training module in green, and the graphical user interface in yellow.

Measurements of the trocar orientation, instrument insertion-depth, and applied force were acquired using an Arduino data acquisition board model “UNO” (Arduino, Ivrea, Italy). Signals were obtained with the use of their corresponding open source library. Figure 2 depicts the flowchart of the tip position and applied force measurement and data processing.

The orientation angles and depth distance measurements were sent through serial port to the host computer for further analysis. A complementary filter was used to overcome the inaccuracies of the gyroscope in the long term and those of the accelerometer in the short term [29]. From the rotation angles and insertion depth data, the tip of the instrument can be tracked by applying rotation matrices. The laparoscopic instrument was initially assumed to be in the vertical position {0} (R_x_, R_y_ = 0°) at a determined depth by an initial reading of the VL53L1X sensor offset by the length of the laparoscopic instruments. Equation (1) computes the insertion depth (P′):

P′ = tool length (cm) − VL53L1X (cm) + 2 cm(1)

Thus, tool-tip position (P) to {0} is computed as shown in Equation (2):

P = R_x_(α) * R_y_(β) * P′ (2)

Furthermore, position filtering based on two subsequent point variations was designed as follows. Experimental results show that the change of P is greater in the z component compared to both x and y components, at each data collection iteration. Hence, the filter neglects data with large variation in the z component. To complement the filter, it was decided to be selective in the minimal distance between the last P point and each P point registered. Both observations were done while the tool was not moving and in the vertical position. A repeatability test was carried out to validate the accuracy of the measured distances. Lines of 1 to 11 cm were drawn in the x–y plane and gauged via a digital caliper model Absolute Digimatic with resolution of 0.01 mm (Mitutoyo^®^, Kanagawa, Japan). Ten measurements were needed to obtain the mean standard deviation of the system.

**Figure 3:**
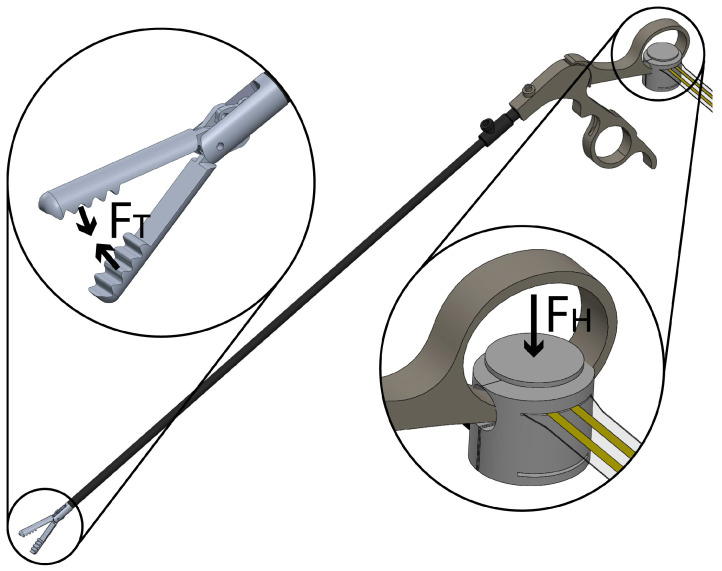
Gripping force sensing module measurement principle. F_T_ represents the real force at the tip. F_H_ represents the force applied at the handle adaptor.

Regarding the force data, the applied forces at the sensors used in the gripping force module and in the phantoms were obtained by measuring a voltage divider output. The thin-film sensors used were calibrated with weights ranging from 0 to 1.95 kg. in increments of 0.05 kg and following the manufacturer’s recommendations for calibration [30]. For the approximation of the gripping force (F_G_), a provisional force-sensing set up was implemented at the tool-tip to obtain data sets of handle force (F_H_) and tool-tip (F_T_) while closing the instrument. The measurement principle is shown in Figure 3. Later, a correlation was done via a linear regression model. During the calibration process, it was found that tip force values started being detected at a certain handle force. Hence, values under this threshold are neglected. In the case of the force training module characterization, phantom force from embedded calibrated sensors, and gripping force module data were plotted to identify differences stiffness, as stated back in Section 2.3.

The initial position and force calculations were conducted within the Arduino environment. Processing was used for further data manipulation and visualization. Once all measurements were processed, the software saved the 3D spatial position of both instrument’s tip, the measured force in all the sensors, and the summary of the user execution parameters: time (s), the total traveled distance of the surgical instrument tip in both left and right hand (cm), mean speed of each surgical instrument tip (cm/s), as well as maximum and mean force (N).

### 2.5. Graphical User Interface Module

**Figure 4:**
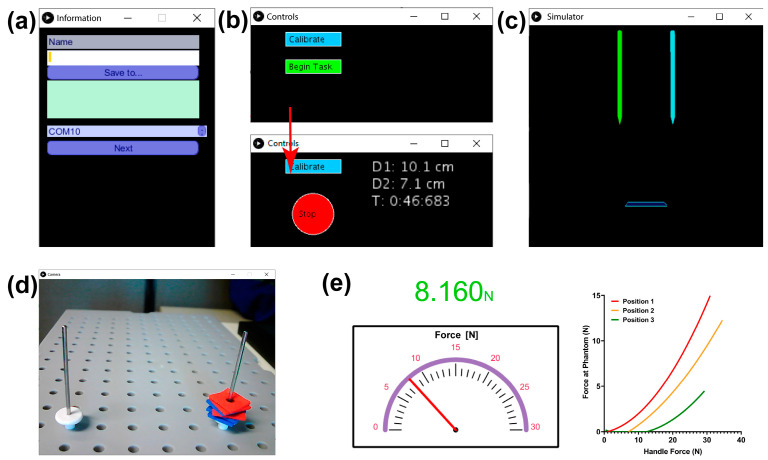
Graphical User Interface Module. (**a**) Initial window for entry user’s identification. (**b**) Control and calibration window. When the task has begun, the control window shows both tools tracked distance and elapsed time. (**c**) Digital twin of both tools. (**d**) Live video from webcam. (**e**) Grasping force window with numeric and graphical indicators and phantom-to-handle force graph with colors and numbers corresponding to carousel position.

To provide users with objective and quantitative information regarding the force applied over a tissue and surgical instrument tracking motion, data are manipulated using libraries in the open-source Processing environment (processing.org, The Processing Foundation). The Graphical User Interface (GUI) is divided into five different windows as shown in Figure 4. First, a form collects identification data from the user (Figure 4a) next, Figure 4b–e appear simultaneously. In the “control” window (Figure 4b), the user must calibrate the position module sensors by locating the instruments vertically, with the help of the training tasks grid, and press the “calibrate” button. A graphical 3D representation of the laparoscopic instruments is shown in “simulator” window (Figure 4c), the user can confirm the correct calibration of the position sensors when the digital instruments are vertical and still. Additionally, they are intended to improve the perspective of the user. Once calibration is done, and the task is initiated, information about elapsed time and distance traveled for each tool is offered numerically in the same window. To initiate the test, the user would need help from another person to manage the interface. At the same time, a third window (Figure 4d) shows live video of the executed task from the webcam, as visual feedback found commonly in practice (Task 1 set up can be seen in Figure 4d). Force feedback window (Figure 4e) depicts the real-time gripping force module measurement, with a numerical indicator changing in color as force increases. Next to it, a graph (phantom force vs. gripping force) of the interaction with phantoms is shown to detect the phantoms based on their stiffness.

### 2.6. Validation in Medical Training Context

**Figure 5:**
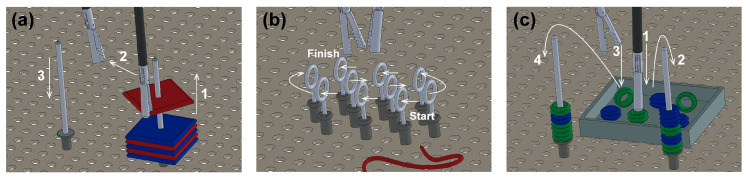
Designed tasks for the validation in the medical training context. (**a**) Peg transfer, (**b**) object transfer, (**c**) pea on a peg.

The functionality and fidelity of the training simulator were validated through 39 subjects including attending surgeons, general surgery residents, and students from our medical school. They executed a series of adapted tasks reported in past works as training assessment exercises [27,31], while control, simulator and live video windows (Figure 4b–d)) of the GUI were displayed on a laptop. Figure 5 shows a graphic representation of the training tasks:

- Peg transfer: the subjects must grip one foam square from a right peg with the right instrument. Once the object is taken out from the peg, the subject must transfer it to the left instrument. Finally, the object must be delivered into the left peg. If a foam square falls, the subject must grip it with the same instrument being used. The task should be done for each of the six (Figure 5a).
- Object transfer: a thread must be inserted through ten rings in a zig-zag array, without any instrument use restriction. The task must be initiated in the right ring nearest to the user and continue horizontally to the two left consecutive rings. Next in the second line, beginning in the left ring and advancing to the right. Finally, in the farthest line, execute like in the first row (from right to left) (Figure 5b).
- Pea on a peg: small plastic tubes must be gripped from a box and be inserted in a peg, alternating between the right and the left one. For the right peg, the pipes must be taken with the right instrument. Likewise, for the left peg, the pipes must be taken with the left instrument. In the end, seven tubes must be inserted into each peg. The color of the gripped object is not important (Figure 5c).

**Figure A1:**
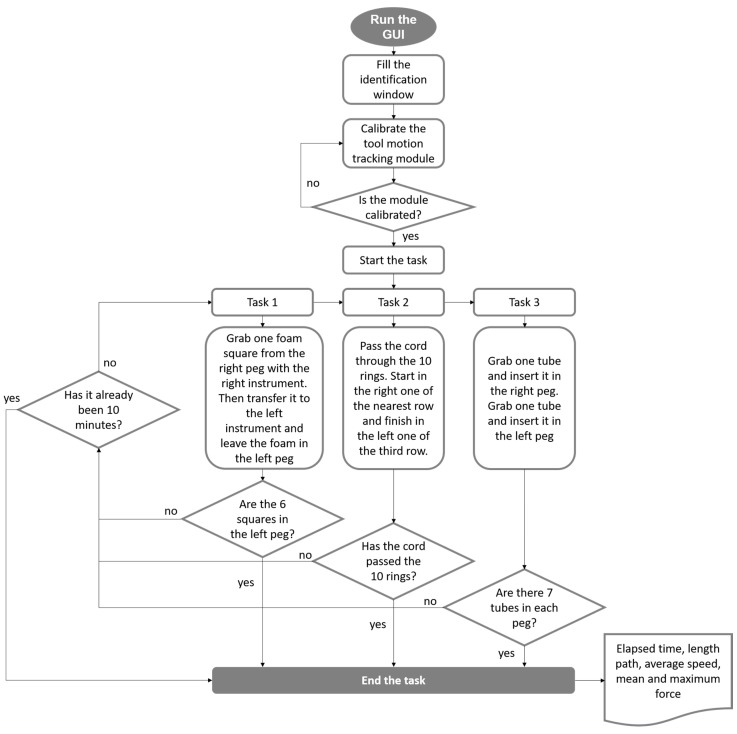
Protocol for validation in medical training context.

The selected tasks validate hand-eye coordination, bimanual dexterity, depth perception, and interaction of the dominant and non-dominant hand, besides the use of different geometries plays a role in the applied grasping force [31]. The tasks instructions were verbally explained to the subjects who volunteered to participate. Participants were divided into three groups with varying levels of experience. The metric for expertise was defined as the number of cholecystectomies performed per year by a given surgeon, yielding the following categories: (a) novices (n ≤ 30), (b) intermediate (30 < n < 50), and (c) experts, (n ≥ 50) [31,32,33]. During execution, surgical instrument motion tracking and applied force were measured and recorded simultaneously. All data were codified to unable the participant’s identification. Following the FLS guidelines, subjects were given a time limit of 10 min. The Figure A1 in the Appendix A shows the flowchart of the training task protocol.

### 2.7. Statistical Analysis

Tool position and grasping force measurements were statistically analyzed. Accuracy in track measurements was tested by a two-sample *t*-test for different distances. Grasping force approximation was validated with linear regression, and differences with real grasping force were tested by a *t*-test. Moreover, differences among phantoms stiffness were validated by an ANOVA and Tukey’s test at a common handle force. Sample measurement repeatability was examined by a polynomic regression of 10 grasping cycle data. Finally, consistency in phantom differentiation was tested in all six possible phantom positions in the carousel. *p*-values lower than 0.05 were considered significant. The results collected from the tasks performed by volunteers were analyzed via the Kruskal-Wallis test and Dunn multiple comparison test. Significance of ten and five percent was analyzed.

## 3. Results

### 3.1. Tool-Motion Tracking Module

**Figure 6:**
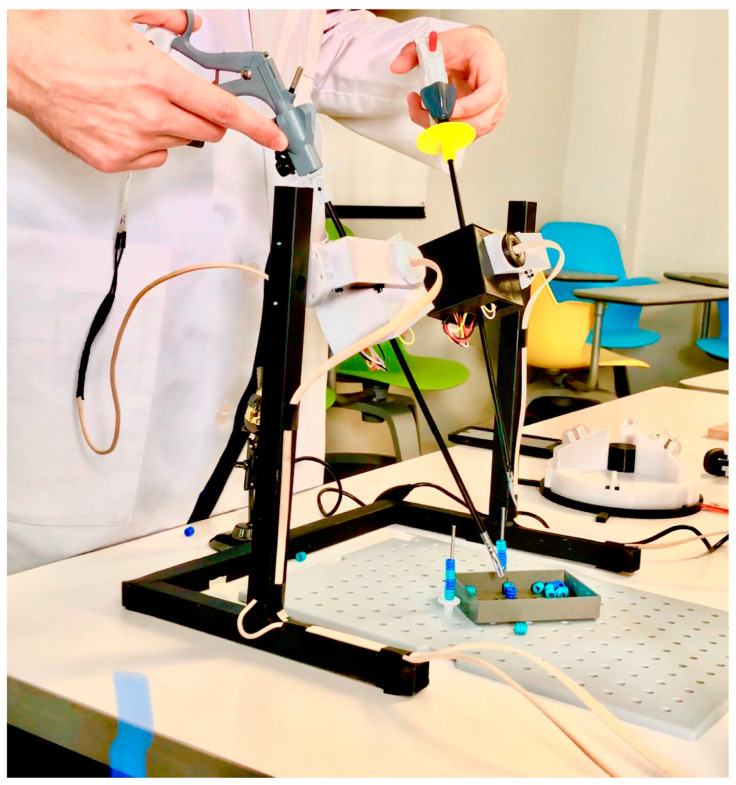
Developed system instrumented with force and motion-tracking sensors during the proposal validation with the use of common laparoscopic instruments.

**Figure 7:**
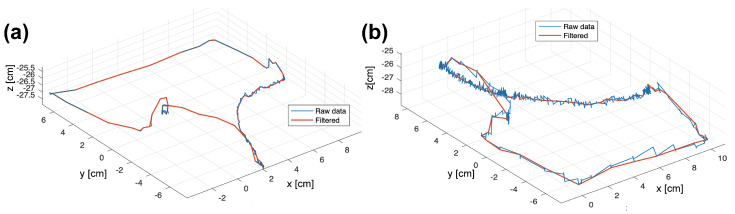
Performance of the noise filter at different motion speeds: (**a**) fast motion rate and (**b**) slow-motion rate.

After several design and prototyping iterations, the final simulator assembly is shown in Figure 6. The motion tracking data tends to generate noise under slow and fast speed motion conditions. Therefore, a complementary filter was used, along with point-to-point speed and Euclidean distance discrimination to smooth data lines. It was detected that, when the tool was not moving, the speed in the z-direction is greater than in the x and y-axis. Thereby, the speed-based filter neglects position data sets when the speed in the z component is greater or equal to 0.005 cm/s, this low value ensures that only points with a no-change position tendency between each iteration are registered. Similarly, it was decided to record minimally separated points by 0.31 cm. The points that meet the conditions of the filter generate a smooth path (shown in Figure 7).

A repeatability test was carried out to validate the distance measurements of the system. The mean standard deviation resulted in 0.094 cm. The data of the repeatability error computed a coefficient of determination of 0.99. Moreover, differences between distances from a caliper and module measurements resulted in non-significant (*p* > 0.05).

### 3.2. Gripping Force Sensing Module

The observed error in the force measurements during the sensor calibration, for both applications in this project, is similar to the one specified by the manufacturer (~3%). Grasping force approximation resulted in a linear relationship of 0.5354 N at the tip per each Newton at the handle, with a correlation factor of 0.9975. Differences between tip approximation and on-site force measurement resulted non-significant (*p* > 0.05). The outcome validates the accuracy in grasping force approximation with the handle force measurement. According to obtained values, the maximum grasping force possible to measure by the sensor is 69.55 N with a resolution of 71.83 mv/N.

Additionally, during the module approximation, it was possible to observe that for handle values lower than 0.950 N, force at the tip was zero Newtons. Forces above this value in the handle were recorded.

### 3.3. Force Training Module

**Figure 8:**
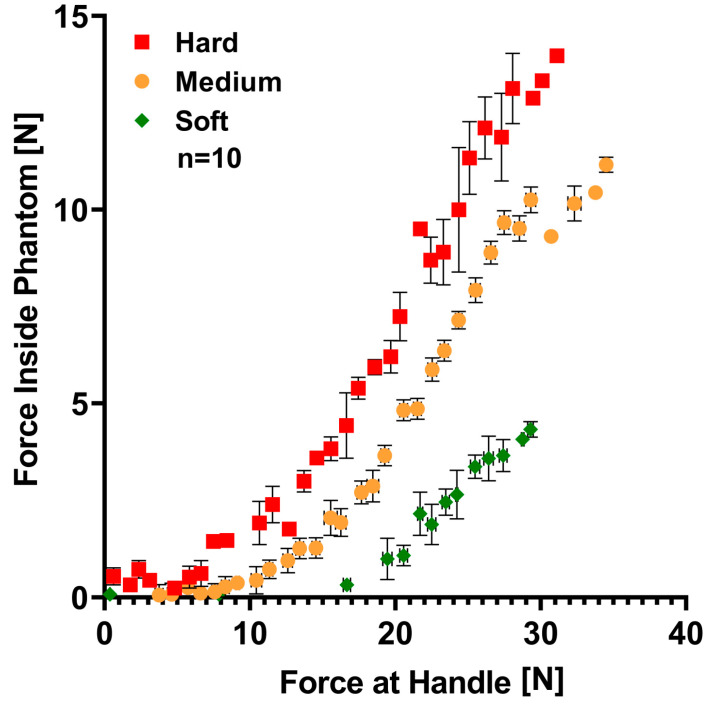
Phantom characterization curve. Each point represents the mean force value of handle force and force inside the phantom with standard deviation bars in both axes after 10 grasping cycles. Red, yellow, and green represent hard, medium, and soft phantoms, respectively.

Different mixing ratios were used to manufacture hard, medium, and soft PDMS phantoms. Resulting differences in phantoms are shown in Figure 8, where the average gripping force module and phantom force of ten measurements, along its respective standard deviation, are graphed. This different behavior is due to the variation in stiffness [34]. Thus, as stiffness increments, phantom deformation decreases when a load is applied over it. Consequently, the transmitted force is higher, measuring greater values inside the phantom; opposite behavior is observed to softer samples, where forces sensed inside phantom are smaller for the same load. Differences in phantoms resulted significant (*p* < 0.01).

Additionally, measurements are considered constant and repeatable with determination coefficients around 95%. Moreover, all six phantom position combinations around the carousel were tested. Results showed the same curve order for all combinations. Data analysis confirms present and clear mechanical properties differences among phantoms, in concordance with Seghir et al. Results confirm the possibility to transfer the training system to a medical context.

### 3.4. Validation in Medical Training Context

**Figure 9:**
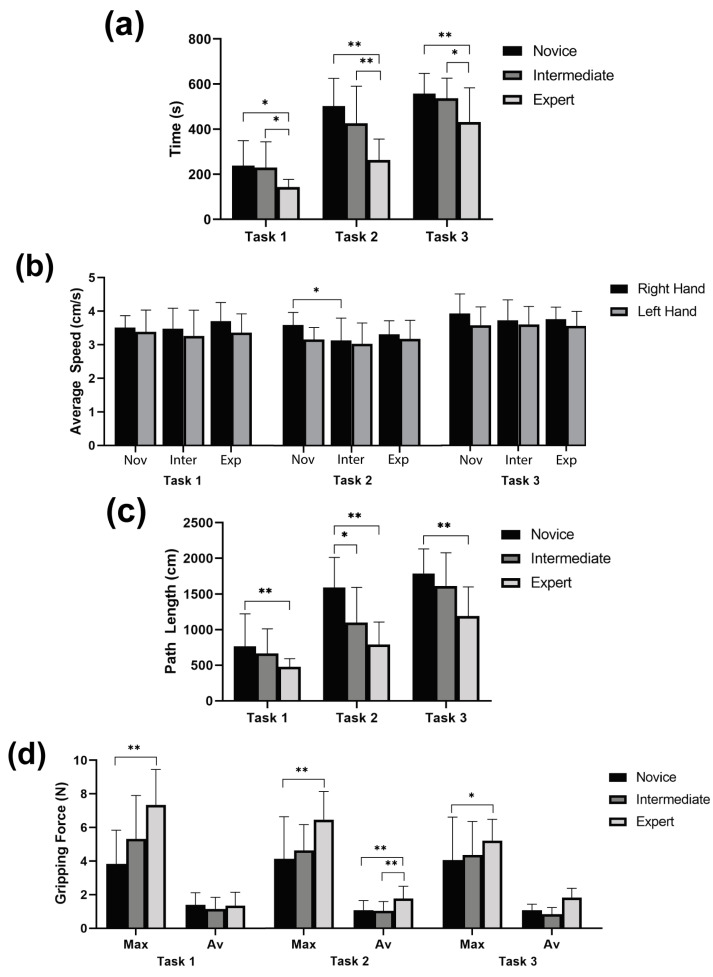
Results of the training tasks (N = 39, bars represent mean ± standard deviation, asterisks represent statistical significance * *p* < 0.1 ** *p* < 0.05). (**a**) Total time needed to complete each task. (**b**) Average speed of both instruments (left and right hand) during the tasks. (**c**) Total distance covered by both instruments (left and right hand). (**d**) Average and maximum grasping force applied during the tasks.

Subjects performed three established training tasks, while time, instrument tip motion tracking, and force data were simultaneously recorded. The corresponding statistical analysis is reported in Figure 9a depicts the total time it took users to complete each task. As expected, compared to other groups, experts complete these tasks in a shorter time. Figure 9b shows the average speed of the instrument tip. Based on this metric, no statically significant difference is found among the different groups. Figure 9c shows the total traveled distance of the instrument tip (adding data for both right and left hands). As expected, by the degree of experience, the novice group tends to follow a longer path to complete the tasks. For the case of intermediate experience, there is no statistically significant difference with this metric. Finally, Figure 9d presents the maximum and average applied gripping force. In this case, the maximum force seems to be a good parameter to distinguish novice vs. expert, with consistent statistical difference in all three tasks.

## 4. Discussion

Several design and prototyping iterations were necessary to complete a functional prototype of the intended modular surgery simulator, with instrument motion tracking and force feedback modules. The system developed by our group was designed to maintain the original laparoscopic instrument functionality, thus being imperceptible to the surgeon’s use. The design of the whole system was inspired by different commercial training boxes, which resembled the working zone where the surgeon executes the surgeries. Though this first version of LAPKaans does not isolate the training area from the subject sight, further steps must include the design of a shielding case for a training experience closer to reality. This proposal is a clear example of the result of a close interdisciplinary collaboration between health sciences and engineering to address surgical drawbacks and medical needs with industrial products and digital solutions.

In this field, several research groups have proposed concepts to aid surgeons to excel at their surgical procedures by means of simulator training. Some developments measure the applied grasping force and/or track the instrument tip motions. However, most of the related work shows concepts with significant complexity compared to the work reported here. These added elements of complexity to track motion involve: (a) use of sensors attached to the instrument´s tip that distort the original design of the laparoscopic instrument [35,36,37,38,39,40]; (b) use of complex systems that require special calibration or the placement of the sensors in a specific location of the room [41,42]; (c) placement of elements on the surgeon’s body [43,44]. These specific locations of the systems may impede proper tool motion and positioning [40]. In the virtual and augmented context, the advanced intelligent systems mainly include actuation, visual imaging, position and force sensing, which enhances the training experience; however, drawbacks are the high cost associated with equipment investment and maintenance, as well as content development, thus limiting the availability [45].

Regarding force measurement, most previous implementations measure forces along the instrument´s shaft [46,47,48,49,50,51], since it transmits the mechanical energy exerted by the surgeon towards the instrument’s tip. Other investigators place the sensor at the instrument´s tip [17,24,52], near the handle [36,53,54], or do not focus their sensing system on the instrument, but to another component, such as the trocar [55] or surgeon’s gestures [56], keeping the original design of the laparoscopic instruments intact. The farther the sensing components are from the tip, the greater the benefits in terms of cleaning, sterilization, and dimensions, but the higher chances for measurement errors [12]. Concerning sensors, most of the systems are based on strain gauges and thin-film sensors. However, novel approaches have proposed the use of optical sensors [49,52].

Our system can track the position of the instrument’s tip, and it is compatible and adaptable to different laparoscopic instruments. The system is portable, the calibration process is easy, and it has a good numerical feedback system. Besides the low cost is due the instrumentation with affordable sensors and manufacturing processes. Moreover, it does not modify the surgical devices and it is completely external to the instrument. LAPKaans features four of the six desirable DOF: three (translation, pitch, and yaw axis) from the designed system, and one (roll axis) in the instrument’s mechanics. Though previous researches have correlated the importance of the roll axis rotation angle in suturing practices [57], this DOF is not measured in the proposal since it does not represent a component in the position of the tip.

The gripping force module is reliable, easy to manufacture, and has a simple calibration procedure. Furthermore, it only occupies about 20 percent in the finger hole of the instrument’s handle. The decision to place the force sensing module at the handle is justified by its benefits in dimensions and sterilization [12,14]. The material used for the handle adaptor is apt for hospital sterilization [58], and sensor material is ideal for applications in high temperature and humidity environments [30]. These features give us the base to think in potential applications for force measurements during surgery at the operating room. However, the gripping force measurements are valid only for grasper closing action forces (compressive force). Additionally, an inaccurate performance is present during small force values (<1 N) measured by the module, since it is not clear whether the detected forces are interactions at the tip or energy needed to activate the instrument mechanism. Thus, values under this threshold are neglected. Further work is needed. The gripping force measurements could be used to offer tactile feedback to the user, in addition to the visual one offered in the developed graphical interface. In literature, the importance of this feedback is reported during the training process [59]. For the development of this feature the design and implementation of a different tool handle and a mechanic grasping system, which emulated the force interaction at the tip would be needed. Nevertheless, this proposed change does not meet the main leading objectives, which are maintaining the original laparoscopic instrument design and keeping the handling as simple as possible. Further work for the implementation of haptic feedback in a simple manner is needed.

The proposed force training module helps surgeons distinguish among samples with different stiffness. The use of phantoms with a difference in stiffness was seen in previous work [59], which detected it by correlating the gripping force and the closing-angle of the tip. Nevertheless, herein we propose the concept of measuring the force from an outer-tool component with the instrumentation of phantoms themselves. Further work is needed to detect differences in stiffness by laparoscopic instrument itself.

The validation in the medical context was conducted under a relevant sample of experienced surgeons, surgery residents, and medical students who volunteered to participate. While we did set a target for medical validation, a large validation process is a future metric to evaluate. Results clearly showcase the capability of the proposed simulator to distinguish the different levels of expertise in laparoscopic exercises. The expert group has developed better spatial awareness and tends to economize their movements with less length path when compared to the novice. This finding is consistent with previous works related to medical training [11,23,60]. Furthermore, contrary to expected and reported in the literature [31,46], the novice group tends to exert less force than the expert group. This may be due to the location of the force sensing system, representing that the expert group has a more constant and better instrument handgrip, resulting in higher maximum and average force values. Further research into this issue is needed.

The use of standard surgical instruments in laparoscopic surgery training, together with grasping force sensing and instrument tip tracking capabilities, opens the door to new education protocols. Currently, training decisions are commonly only based on the execution time of training activities as the key parameter to decide whether a surgical trainee is capable or not to perform a real surgery. New parameters must indicate real-time or post-training objective information, as the gripping force, the accumulated distance, or the instrument motion speed, and thus standardize the level of surgeon skills.

**Figure 10:**
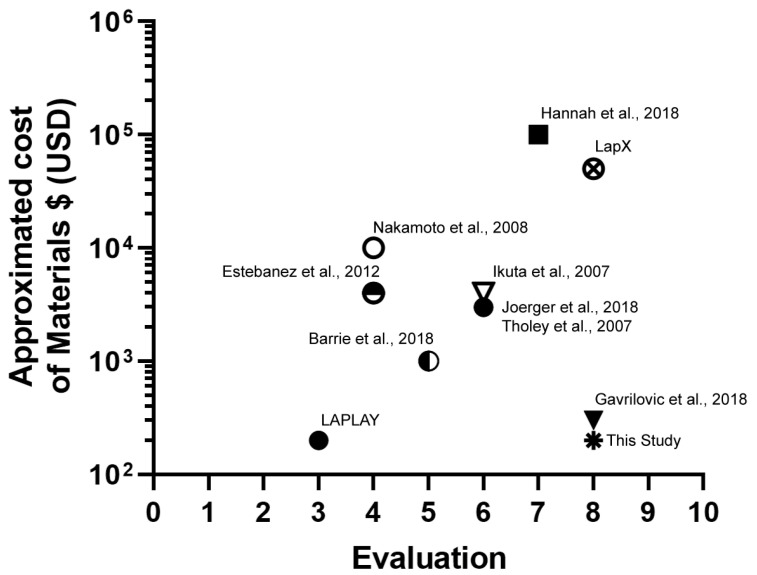
The cost-benefit graph for our proposal compared with commercial and academic approaches.

To compare the affordability of the proposed simulator, Figure 10 provides a cost-comparison to a wide range of training simulators currently in the market and research prototypes. The “current study” refers to the cost of our simulator components (not including the standard laparoscopic surgery instruments). We considered the commercial systems Lap-X (Medical X, Rotterdam, The Netherlands) and LaPlay (Jinan One Half Industrial Design, Shandong, China) box trainers with haptic force feedback and no force feedback, respectively; as well as a proposal from Tavakoli et al., a system based in photonic crystal fiber sensor; proposals of Barrie et al. and Hannah et al., both based on force sensing at the handle; and Tholey’s proposal, with the grasper-tip instrumented and hydrogel phantoms [13,14,53,59,61,62]. Moreover, some researchers employ specialized and costly devices to measure the position and orientation of the laparoscopic instrument. Other researchers use specialized software for computing the instrument’s position [35,42,63], and an approach with similar features made by Gavrilovic et al. [55]. A benchmark comparison was conducted qualifying each system in a scale from 1 to 10 based on desired and useful features. The reference features used in this benchmark comparison are the following: (a) compatibility with standard surgical instruments, (b) need to modify surgical instruments, (c) portability, (d) need for wearable devices, (e) compatible with real surgery, (f) 4 DOF, (g) visual feedback, (h) haptic feedback, (i) surgical instrument tip tracking, (j) tracking of gripping force.

Though the LAPKaans system is simple in use and portability as a basic training box, the laparoscopic training experience is enhanced by the offered execution information. Approaching to augmented-reality (AR) or virtual-reality (VR) simulator, but at a lower cost.

## 5. Conclusions

Laparoscopic surgery is a minimally invasive technique that has been the gold standard in the last decades, in many procedures, thanks to its benefits to patients. Nevertheless, a high-performance level is required and the surgeon deals with a long learning curve. Despite this, there is a wide range of laparoscopic surgery trainers, but just a few of them offer objective feedback.

Therefore, we present a low-cost (<USD $200 in components) and adaptable training system capable of tracking the instrument-tip’s motion and the grasping force applied. This system can be used with any tool since components are detachable and external to the instrument. Results showed a position mean deviation of 0.094 cm and a force approximation correlation of 0.9975, though small forces (<1 N) cannot be accurately measured. Moreover, we developed a training system based on PDMS sensorized phantoms with clear and proven differences in stiffness, which emulate different tissue types.

With the developed system, differences in expertise performance among novice, intermediate, and expert subjects were detected regarding tracked distance, average, and maximum force. This premise opens the door to new training protocols. Objective parameters that take an important role in trainee qualification.

Future work is needed to make the training system more robust by isolating the working zone from user sight. Next steps are mandatory to transfer the measurement of the gripping force to a real clinical environment. The grasping force sensing module should be improved to accurately differ interaction forces at the tip, from the force applied in the handle when closing the grasper, as well as sample stiffness differentiation by the tool itself, with an additional tool parameter further than the grasping force, and the adaptation of phantom materials with the properties of living tissues. Finally, a larger validation in a clinical context should be held to properly justify the use of the new low-cost training system in new training assessments.

## Appendix A. Protocol for Validation in Medical Training Context

The following flowchart provides more detail in regards to the protocol for validation with surgeons.
